# *In silico* studies on leishmanicide activity of limonoids and fatty acids from *Carapa guianensis* Aubl

**DOI:** 10.3389/fchem.2024.1394126

**Published:** 2024-07-30

**Authors:** Renilson Castro de Barros, Renato Araujo da Costa, Suelem Daniella Pinho Farias, Kelly Cristina Oliveira de Albuquerque, Andrey Moacir R. Marinho, Marliane Batista Campos, Patrícia Santana Barbosa Marinho, Maria Fani Dolabela

**Affiliations:** ^1^ Pharmaceutical Sciences Postgraduate Program, Federal University of Pará, Belém, PA, Brazil; ^2^ Federal Institute of Education Sciences of the State of Pará, Abaetetuba, PA, Brazil; ^3^ Faculty of Pharmacy, Federal University of Pará, Belém, PA, Brazil; ^4^ Biotechnology and Biodiversity Postgraduate Program (BIONORTE), Federal University of Pará, Belém, PA, Brazil; ^5^ Post-Graduation in Chemistry, Federal University of Pará, Belém, PA, Brazil; ^6^ Leishmaniasis Laboratory, Evandro Chagas Institute, Ananindeua, PA, Brazil

**Keywords:** methyl angolensate, 6-hydroxy-methyl angolensate, arachidic acid, myristic acid, COX-2, hypoxia-inducibke factor 1 alpha

## Abstract

The oil of *Carapa guianensis* showed leishmanicidal activity, with its activity being related to limonoids, but fatty acids are the major constituents of this oil. The present study evaluated the physicochemical, pharmacokinetic, and toxicity profiles of limonoids and fatty acids already identified in the species. Based on these results, 2 limonoids (methyl angosinlate, 6-OH-methyl angosinlate) and 2 fatty acids (arachidic acid; myristic acid) were selected for the prediction of possible targets and molecular docking. Included in this study were: Gedunin, 6α-acetoxygedunin, Methyl angosenlato, 7-deacetoxy-7-oxogedunin, Andirobin, 6-hydroxy-angolensate methyl, 17β-hydroxyazadiradione, 1,2-dihydro-3β-hydroxy-7-deacetoxy-7-oxogedunin, xyllocensin k, 11beta-Hydroxygedunin, 6α,11-11β-diacetoxygedunin, Oleic Acid, Palmitic Acid, Stearic Acid, Arachidic Acid, Myristic Acid, Palmitoleic Acid, Linoleic Acid, Linolenic Acid, and Beenic Acid. Regarding physicochemical aspects, fatty acids violated LogP, and only limonoid 11 violated Lipinski’s rule. A common pharmacokinetic aspect was that all molecules were well absorbed in the intestine and inhibited CYP. All compounds showed toxicity in some model, with fatty acids being mutagenic and carcinogenic, and limonoids not being mutagenic and carcinogenic at least for rats. In in vivo models, fatty acids were less toxic. Molecular dockings were performed on COX-2 steroids (15 and 16) and hypoxia-inducible factor 1 alpha for limonoids (3,6), with this target being essential for the intracellular development of leishmania. Limonoids 3 and 6 appear to be promising as leishmanicidal agents, and fatty acids are promising as wound healers.

## 1 Introduction

The treatment of leishmaniasis is carried out using pentavalent antimonials, which are chemotherapeutic agents of high cost, requiring long-term treatment and capable of causing strong adverse reactions that negatively interfere with treatment adherence (Mann et al., 2021). Another drug is Amphotericin B (Aguiar and Rodrigues, 2017), which also presents similar problems to antimonials, being a high-cost and highly toxic treatment (Mcgwire and Satoskar, 2014; Falci and Pasqualotto, 2015).

Another issue related to leishmanicidal drugs is the increasing parasite resistance, which makes it necessary to search for pharmacological alternatives (Rodrigues et al., 2006). Andiroba oil (*C. guianensis*) is used by traditional communities for the treatment of wounds (Pinto, 1963). From *Carapa guianensis* oil, limonoids have been identified, with the main ones highlighted as: gedunin, 6α-acetoxygedunin, methyl angolensate, 7-deacetoxy-7-oxogedunin, andirobin, 6-hydroxymethyl angolensate, 17β-hydroxyazadiradione, 1,2-dihydro-3β-hydroxy-7-deacetoxy-7-oxogedunin, and xylolcensin K (Ambrozin et al., 2006; Tappin et al., 2008; Silva et al., 2009). The metabolites in higher concentration are fatty acids (palmitic and oleic acid), followed by stearic, linoleic, linolenic, myristic, palmitoleic, and behenic acids (Salgado et al., 2015).

The seed oil of *C. guianensis* showed no antileishmanial activity, and the cytotoxicity was higher than 1,000 μg/mL against peritoneal macrophages. The limonoid-rich oil fraction demonstrated activity against promastigotes *Leishmania amazonensis* (IC_50_ = 10.53 μg/mL), amastigotes (IC_50_ = 27.31 μg/mL), and exhibited cytotoxicity (IC_50_ = 78.55 μg/mL) (Oliveira et al., 2018). In summary, the leishmanicidal activity may be related to the limonoids; however, there is a lack of data on the physicochemical, pharmacokinetic aspects, and possible mechanism of action. On the other hand, the major compounds of *C. guianensis* are fatty acids, and studies on these compounds are limited.

Using predicton methods, this work reports on the physicochemical properties, pharmacokinetics, toxicological aspects, potential activities, and targets involved of limonoids and fatty acids identified in *C. guianensis* oil, as well as their potential mechanisms of action involved in leishmanicidal activity.

## 2 Materials and methods

### 2.1 Criteria for the selection of molecules

The following limonoids were selected: gedunin, 6α-acetoxygedunin, methyl angolensate, 7-deacetoxy-7-oxogedunin, andirobin, 6-hydroxymethyl angolensate, 17β-hydroxyazadiradione, 1,2-dihydro-3β-hydroxy-7-deacetoxy-7-oxogedunin, xylolcensin K (Ambrozin et al., 2006; Tappin et al., 2008; Silva et al., 2009), 11beta-Hydroxygedunin, and 6α,11β-diacetoxygedunin (Oliveira et al., 2018).

The following fatty acids were also selected for prediction studies: oleic acid, palmitic acid, stearic acid, arachidic acid, myristic acid, palmitoleic acid, linoleic acid, linolenic acid, and behenic acid (Salgado et al., 2015; Silva, 2018).

### 2.2 In silico evaluation

The molecules were drawn using the Marvin (2023) online program (https://marvinjs-demo.chemaxon.com/latest/demo.html), and for the determination of physicochemical properties, the online server Home-ADMElab was used (https://admet.scbdd.com) (Dong, 2024). The Lipinski’s Rule of Five or “Rule of Five” was considered (Lipinski, 2004). For pharmacokinetic and toxicity predictions, the PreADMET program (version 2.0, Copyright ^©^ 2005–2017) was used, which considers pharmacokinetic properties (A–absorption; D–Distribution; M–Metabolism/Biotransformation; E–Excretion) and evaluation of toxicity parameters (T–Toxicity; Preadmet, 2020).

For the assessment of toxicity in marine organisms, the criteria used were as follows: for toxicity in algae (Costa et al., 2008); for Daphnia sp (Guilhermino et al., 2000); for Medaka (Zucker, 1985); and for Minnow (Costa et al., 2008). The mutagenicity risk was assessed by the Ames test with the following strains of *Samonella Typhimurium:* TA100-10RLI and TA 100-NA mutation in His G46e plasmid pKM101 without S9; TA1535- 10RLI and TA1535-NA mutation in His G46 (Ames et al., 1975). The carcinogenic potential of the compounds was evaluated in rats and mice and referred to as (+) carcinogenic and (−) non-carcinogenic. To predict acute oral toxicity (lethal dose 50%- LD_50_), the online software PROTOX II was used (Drwal et al., 2014), considering the classification from I to VI, according to ABNT NBR 14725-2 (2019). Adverse events that may occur with the use of the molecule were also evaluated.

The search for potential targets for molecular docking prediction was conducted using the SuperPred Webserver program (Nickel et al., 2014), a server for predicting molecular targets with potential interaction with the investigated ligands. The targets, which showed relevance to the investigated biological activity, were obtained from the Protein Data Bank database (PDB ID 4H6J and 5F19/4OTY). Compounds with the highest scores for therapeutic activity (≥70% probability of binding and ≥70% prediction accuracy) were selected for molecular docking simulations.

### 2.3 Docking molecular

Molecular targets were determined: Hypoxia-inducible factor 1 alpha (HIF-1-α, PDB 4H6J) and Cyclooxygenase-2 (COX-2, PDB 5F19/4OTY). The crystallographic structure of the enzymes was retrieved from the Protein Data Bank (PDB) under the codes 4H6J (Cardoso et al., 2012) with a resolution of 1.52 Å and 4OTY with a resolution of 2.35 Å.

The structures of the compounds were initially obtained from PubChem (http://pubchem.org) in sdf format. OpenBabel (O’Boyle et al., 2011) was used to generate the 3D coordinates of the compounds and optimized using the Gaussian 09 software. Docking molecular simulations were conducted using the program Molegro Virtual Docker (MVD) version 5.5 (Bitencourt-Ferreira and de Azevedo, 2019).

Redocking was performed using the inhibitor lumiracoxib (LUR) of the COX-2 protein (PDB 4OTY). The enzyme’s active site was defined as a spherical region of 12 Å, based on the coordinates of the crystallographic ligand lumiracoxib using the MolDock Score scoring function.

For HIF-1-α, due to the absence of a crystallized inhibitor, data from the literature and the cavity detector of the program (Singh et al., 2023; Kong et al., 2022) and the cavity detector of the MVD with coordinates x: 6.35, y: −26.39, z: −22.37 and a sphere of 12 Å were used. Ligands underwent 10 iterative runs, and the pose with the best scoring result was considered for the analysis of intermolecular interactions using the Discovery Studio Visualizer (Discovery Studio Visualizer Dassault Systèmes BIOVIA, 2021).

### 2.4 Molecular dynamics (MD)

The stability of the ligand-receptor complexes for the apo form of HIF-1alpha and its form complexed with molecules 3, 6, and the reference inhibitor lificiguat (YC-1) was analyzed. Also, the apo form of COX-2 complexed with molecules 15, 16, and the reference inhibitor lumiracoxib. The AMBER22 simulation package was used to perform 200 ns MD simulations on all complexes prepared using the GPU-accelerated version of the Particle Mesh Ewald Molecular Dynamics (PMEMD) (Lee et al., 2018).

Proteins and ligands were prepared in ff14SB (Maier et al., 2015) and GAFF (Wang et al., 2004), with atomic charges calculated using the restrained electrostatic potential (RESP) protocol at the HF/6-31G*25 theoretical level using the Gaussian 09 software. The protonation states of the ionizable residues were analyzed by calculating the pKa at neutral pH using the PDB2PQR server (Dolinsky et al., 2007). All systems were solvated in the tLeap module using an octahedral water box with the TIP3P model (Jorgensen et al., 1983). Na + ions were added to maintain the system’s electroneutrality. Each step was performed by applying steps of steepest descent minimization followed by 5,000 of conjugated gradient.

The systems were heated from 0 to 300 K, maintained at 300 K (Langevin thermostat), performing 200 ps of MD and 300 ps of density equilibration, and 500 ps without positional restraints at constant pressure. A cutoff point of 10 Å for the systems was used for non-bonded interactions, the Particle Mesh Ewald (PME) method (Petersen, 1995), and the SHAKE algorithm (Elber, 2011) were used to restrict bond lengths involving hydrogen atoms. Finally, MD (production) simulations were performed using 200 ns at a temperature of 300 K without positional restraints. The deviations of the protein and protein-ligand complex systems were analyzed by calculating the root mean square deviation (RMSD), root mean square fluctuation (RMSF), and hydrogen bonds using the CPPTRAJ module (Roe and Cheatham, 2013).

### 2.5 Binding free energy calculation using MM/GBSA

The MM/GBSA technique accurately calculates the total binding free energy of protein-ligand complexes using the AmberTools23 package (Da Costa et al., 2022; Case et al., 2023). The last 10 ns of the MD simulation trajectories were used to calculate the binding free energy.

## 3 Results

### 3.1 In silico evalution

**FIGURE 1:**
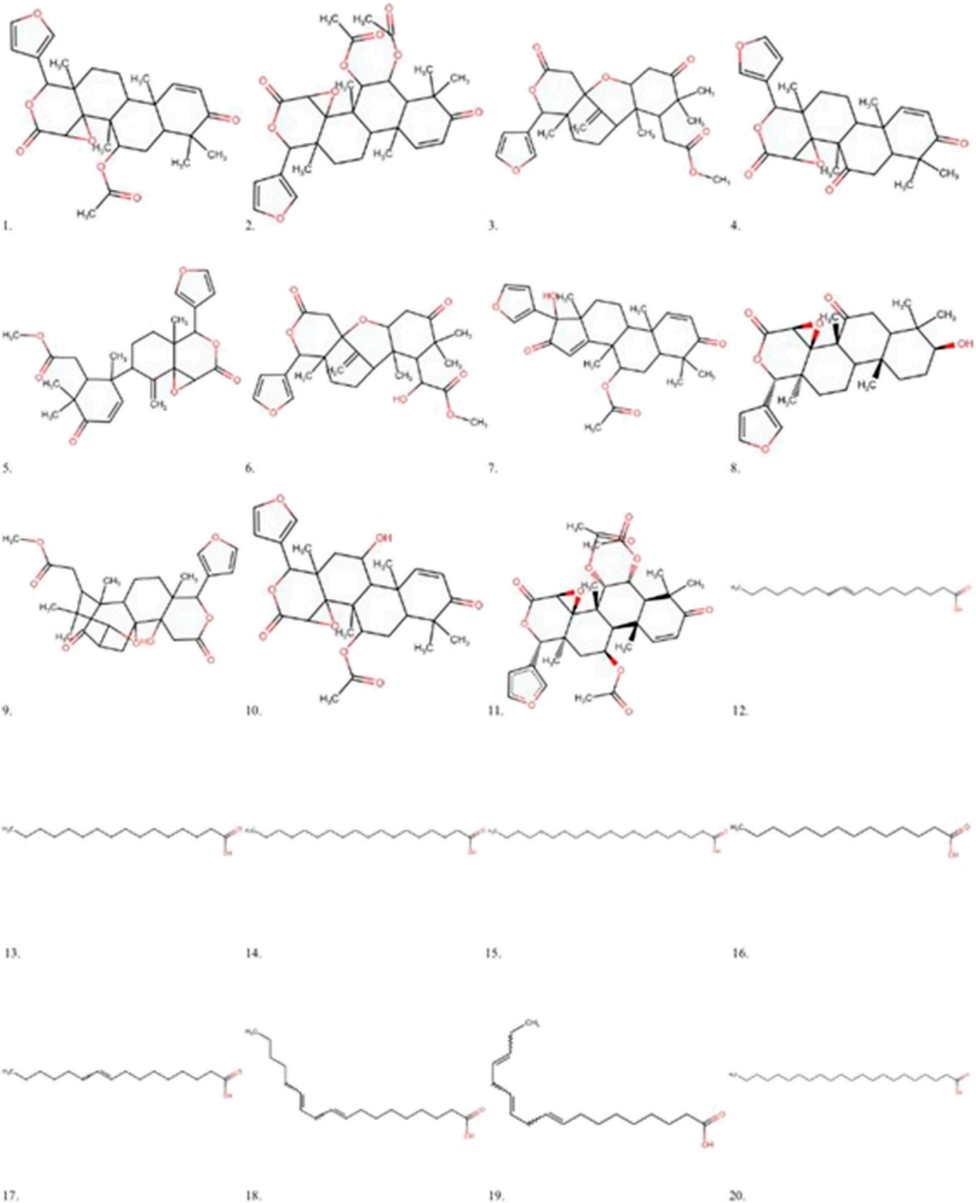
Main Limonoids and fatty acids isolated from *Carapa guianensis* oil. 1 - Gedunin, 2 - 6α-acetoxygedunin, 3 - Methyl angolensate, 4 - 7-deacetoxy-7-oxogedunin, 5 - Andirobin, 6 - 6-hydroxy-methyl angolensate, 7–17β-hydroxyazadiradione, 8–1,2-dihydro-3β-hydroxy-7-deacetoxy-7-oxogedunin, 9 - Xylocensin K, 10–11beta-Hydroxygedunin, 6 α, 11–11β-diacetoxygedunin, 12 - Oleic acid, 13 - Palmitic acid, 14 - Stearic acid, 15 - Arachidic acid, 16 - Myristic acid, 17 - Palmitoleic acid, 18 - Linoleic acid, 19 - Linolenic acid, 20 - Behenic acid.

All limonoids already isolated from *C. guianensis* were included in this study. Similarly, identified fatty acids of the species were selected (Figure 1):

Regarding the predictions of the physicochemical characteristics of the fatty acids (12, 13, 14, 15, 17, 18, 19, and 20), they demonstrated a partition coefficient oil-water (LogP) higher than 5.0, while the limonoids have higher molecular masses (MM), with limonoid 11 violating the Lipinski’s rule. Molecule 2 showed only one violation in molecular mass (Table 1).

**TABLE 1:** Prediction of physicochemical properties.

Molecules	MM	LogP	TPSA	nHBA	nHBD
1	482.57	4.56	95.34	7	0
2	540.00	4.10	121.64	9	0
3	470.56	4.56	92.04	7	0
4	438.52	4.19	86.11	6	0
5	468.54	4.33	95.34	7	0
6	486.56	3.35	112.27	8	1
7	466.57	4.52	93.81	6	1
8	442.55	4.21	89.27	6	1
9	486.56	3.36	112.27	8	1
10	498.52	3.53	115.57	8	1
11	598.64	3.64	147.94	11	0
12	282.46	6.10	37.30	1	1
13	256.43	5.55	37.30	1	1
14	284.48	6.33	37.30	1	1
15	312.53	7.11	37.30	1	1
16	228.37	4.77	37.30	1	1
17	254.41	5.32	37.30	1	1
18	280.45	5.88	37.30	1	1
19	278.43	5.66	37.30	1	1
20	340.59	7.89	37.30	1	1

Lipinski’s rule: LogP - oil-water partition coefficient ≤5; TPSA: topological polar surface area ≤140 Å; nHBA: number of hydrogen bond acceptors ≤10; nHBD: number of hydrogen bond donor groups ≤5; MM, molecular mass ≤500D (Lipinski, 2004). 1 - Gedunin, 2 - 6α-acetoxygedunin, 3 - Methyl angolensate, 4 - 7-deacetoxy-7-oxogedunin, 5 - Andirobin, 6 - 6-hydroxy-methyl angolensate, 7–17β-hydroxyazadiradione, 8–1,2-dihydro-3β-hydroxy-7-deacetoxy-7-oxogedunin, 9 - Xylocensin K, 10–11beta-Hydroxygedunin, 6 α, 11–11β-diacetoxygedunin, 12 - Oleic acid, 13 - Palmitic acid, 14 - Stearic acid, 15 - Arachidic acid, 16 - Myristic acid, 17 - Palmitoleic acid, 18 - Linoleic acid, 19 - Linolenic acid, 20 - Behenic acid.

Despite the compounds’ permeability ranging from low to high, all molecules appear to be well absorbed in the gastrointestinal tract. Regarding distribution, molecules 2, 5, 6, 9, 10, and 11 exhibit reduced plasma protein binding and moderate distribution to the central nervous system (CNS), except molecule 6, which showed low distribution. Only the fatty acids distribute highly to the CNS, likely due to their high lipid solubility (Chagas et al., 2022). All limonoids inhibit CYP2C9 and CYP3A4, with CYP3A4 being the main enzyme involved in the metabolism of these molecules. Fatty acids are inhibited by CYP2C19, CYP2C9, and CYP3A4 and do not undergo phase 1 metabolism (Table 2).

**TABLE 2:** Prediction of pharmacokinetic properties.

		Absorption		Distribution		Metabolism	
Molecules	MDCK	Caco 2	HIA	PP	BBB	CYP Inibition	CYP phase 1
1	L	M	H	S	M	2C9,3A4	3A4
2	L	M	H	F	M	2C9,3A4	3A4
3	L	M	H	S	M	2C9,3A4	3A4
4	M	M	H	S	M	2C9,3A4	3A4
5	L	M	H	F	M	2C9,3A4	3A4
6	L	M	H	F	L	2C9,3A4	3A4
7	L	M	H	S	L	2C9,3A4	CYP3A4
8	M	M	H	S	L	2C9,3A4	CYP3A4
9	L	M	H	F	M	2C9,3A4	CYP3A4
10	L	M	H	F	M	2C9,3A4	CYP3A4
11	M	M	M	F	M	2C9,3A4	CYP3A4
12	H	M	H	S	H	2C19,2C9,3A4	-
13	H	M	H	S	H	2C19,2C9,3A4	-
14	M	M	H	S	H	2C19,2C9,3A4	-
15	M	M	H	S	H	2C19,2C9,3A4	-
16	M	M	H	S	H	2C19,2C9,3A4	-
17	H	M	H	S	H	2C19,2C9,3A4	-
18	H	M	H	S	H	2C19,2C9,3A4	-
19	H	M	H	S	H	2C19,2C9,3A4	-
20	M	M	H	S	H	2C19,2C9,3A4	-

BBB: blood-brain barrier; CYP: cytochrome P450; HIA: human intestinal absorption, S*: strongly; F*: freely; NO: not observed; W: weakly; H: high; L: low; M: medium; 1 - Gedunin, 2 - 6α-acetoxygedunin, 3 - Methyl angolensate, 4 - 7-deacetoxy-7-oxogedunin, 5 - Andirobin, 6 - 6-hydroxy-methyl angolensate, 7–17β-hydroxyazadiradione, 8–1,2-dihydro-3β-hydroxy-7-deacetoxy-7-oxogedunin, 9 - Xylocensin K, 10–11beta-Hydroxygedunin, 6 α, 11–11β-diacetoxygedunin, 12 - Oleic acid, 13 - Palmitic acid, 14 - Stearic acid, 15 - Arachidic acid, 16 - Myristic acid, 17 - Palmitoleic acid, 18 - Linoleic acid, 19 - Linolenic acid, 20 - Behenic acid.

The toxicity prediction model showed a limitation regarding molecule 11, for which it was not possible to determine the toxicity parameters. All compounds were toxic to algae, Daphnia, and Medaka and Minnow fishes. Regarding mutagenicity, the fatty acids were mutagenic for strain TA1535_NA. The fatty acids were carcinogenic for rats and mice. Except for acids 13, 14, 15, and 16, which were not carcinogenic for mice. The limonoids were not mutagenic, but they were carcinogenic for rats and mice, except for 3, 6, and 8, which were not carcinogenic for mice (Table 3).

**TABLE 3:** Prediction of toxicity.

Molecules	Alga	Daphnia	Fish		Ames	Carcino
			Medaka	Minnow		Rats/Mice
1	T	T	VT	VT	N	P/P
2	T	T	VT	VT	N	P/P
3	T	T	VT	VT	N	P/N
4	T	T	VT	VT	N	P/P
5	T	T	VT	VT	N	P/P
6	T	T	VT	VT	N	P/N
7	T	T	VT	VT	N	P/P
8	T	T	VT	VT	N	P/N
9	T	T	VT	VT	N	P/N
10	T	T	VT	VT	N	P/P
11	-	-	-	-	-	-
12	T	T	VT	VT	1535-NA	P/P
13	T	T	VT	VT	1535-NA	P/N
14	T	T	VT	VT	1535-NA	P/N
15	T	T	VT	VT	1535-NA	P/N
16	T	T	VT	VT	1535-NA	P/N
17	T	T	VT	VT	1535-NA	P/P
18	T	T	VT	VT	1535-NA	P/P
19	T	T	VT	VT	1535-NA	P/P
20	T	T	VT	VT	1535-NA	P/P

T: toxic; NT: non-toxic; N: negative; P: positive. Parameters: Algae - < 1 mg/L toxic; >1 mg/L non-toxic (Costa, et al., 2008); Daphnia Test: <0.22 μg/mL toxic; >0.22 μg/mL - non-toxic (Guilhermino, et al., 2000); Test on Medaka and Minnow fish: <1 mg/L - very toxic; 1–10 mg/L-toxic; 10–100 mg/L-harmful and >100 mg/L-extremely toxic (Zucker, 1985), Carcino Rat/mice* = carcinogenicity in rat/mice. T-toxic, NT-non-toxic, VT-very toxic, N-negative, P-positive. 1 - Gedunin, 2 - 6α-acetoxygedunin, 3 - Methyl angolensate, 4 - 7-deacetoxy-7-oxogedunin, 5 - Andirobin, 6 - 6-hydroxy-methyl angolensate, 7–17β-hydroxyazadiradione, 8–1,2-dihydro-3β-hydroxy-7-deacetoxy-7-oxogedunin, 9 - Xylocensin K, 10–11beta-Hydroxygedunin, 6 α, 11–11β-diacetoxygedunin, 12 - Oleic acid, 13 - Palmitic acid, 14 - Stearic acid, 15 - Arachidic acid, 16 - Myristic acid, 17 - Palmitoleic acid, 18 - Linoleic acid, 19 - Linolenic acid, 20 - Behenic acid.

Regarding acute oral toxicity, the molecules with the lowest toxic potential are the fatty acids (Class V and VI); however, despite being considered of low toxicity (Class IV), the limonoids appear to have a lower potential for side effects (Table 4).

**TABLE 4:** Prediction of oral toxicity.

Molecules	LD_50_ (mg/kg)	Toxicity class	Side effects
1	980	IV	N
2	1,004	IV	N
3	846	IV	N
4	596	IV	N
5	1,219	IV	N
6	1,162	IV	N
7	496	IV	N
8	696	IV	N
9	676	IV	N
10	559	IV	N
11	-	-	N
12	5,302	VI	N
13	4,010	V	I/T
14	4,499	V	I/T/M
15	4,867	V	N
16	3,033	V	I/M
17	4,906	V	N
18	5,259	VI	N
19	6,838	VI	N
20	5,228	VI	N

LD50 - lethal dose 50%. NO, nothing observed. I - Irritant, T - Tumorigenic, M - Mutagenicity. Category I: 1< LD50≤ 5 mg/kg - Extremely Toxic; Category II: 5 < LD50 ≤ 50mg/kg- Highly Toxic; Category III: 50 < LD50 ≤ 300 mg/kg - Moderately Toxic; Category IV: 300 < LD50 ≤ 2,000 mg/kg - Low Toxic; Category V: 2000 < LD50 ≤ 5,000 Unlikely to Cause Acute Damage; Category VI: DL50 > 5,000 No damage. Source: ABNT NBR, 2009; RDC, No. 294, 2019. 1 - Gedunin, 2 - 6α-acetoxygedunin, 3 - Methyl angolensate, 4 - 7-desacetoxy-7-oxogedunin, 5 - Andirobin, 6 - 6-hydroxy-methyl angolensate, 7–17β-hydroxyazadiradione, 8–1,2-dihydro-3β-hydroxy-7-desacetoxy-7-oxogedunin, 9 - Xylocensin K, 10–11beta-Hydroxygedunin, 6α, 11–11β-diacetoxygedunin, 12 - Oleic Acid, 13 - Palmitic Acid, 14 - Stearic Acid, 15 - Arachidic Acid, 16 - Myristic Acid, 17 - Palmitoleic Acid, 18 - Linoleic Acid, 19 - Linolenic Acid, 20 - Behenic Acid.

Based on the predictions related to physicochemical, pharmacokinetic, and toxicity parameters, the molecules considered most promising were 3, 6, 15, 16. Subsequently, the targets with potential for biological activity related to Leishmania were determined (Hypoxia-inducible factor 1 alpha, Cyclooxygenase-2) with a probability of correctness and accuracy greater than 70%, and PDB (Protein Data Bank) code (4H6J and 5F19/4OTY) for docking, obtained through the online server as demonstrated in Table 5.

**TABLE 5:** Molecular target assessment.

Molecules	Probability (%)	Prediction accuracy (%)	Target Name	PDB
3	99.05	85.14	Hypoxia-inducible factor 1 alpha	4H6J
6	95.62	85.14	Hypoxia-inducible factor 1 alpha	4H6J
15	90.73	89.63	Cyclooxygenase-2	5F19/4OTY
16	90.93	89.63	Cyclooxygenase-2	5F19/4OTY

PDB: Protein Data Bank 3- Methyl angolensate, 6 - 6-hydroxy-methyl angolensate, 15 - Arachidic Acid, 16 - Myristic Acid.

### 3.2 Docking molecular simulation

**FIGURE 2:**
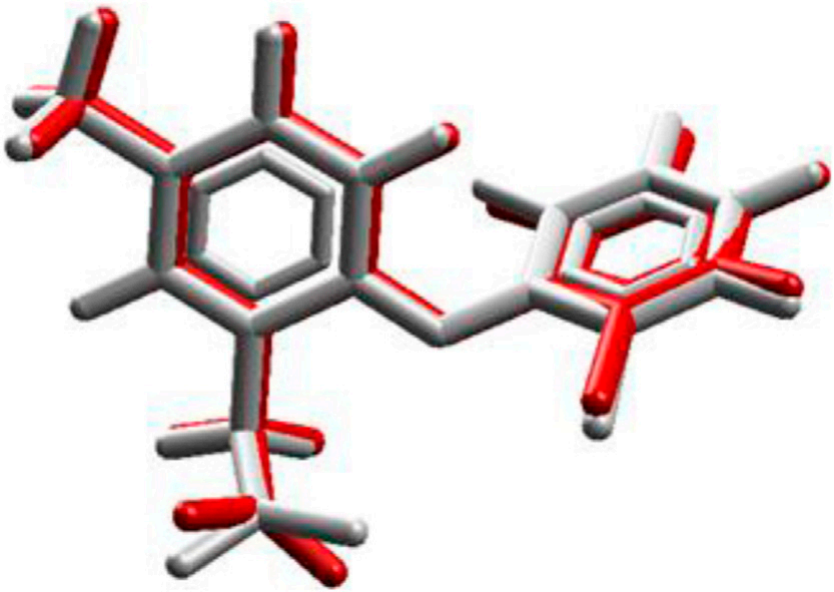
Validation of molecular docking protocols using the MVD program. White is the co-crystal ligand and red is the coupling pose.

In the redocking with the lumiracoxib (LUR) inhibitor of the COX-2 protein (PDB 4OTY), it was found that the redocked conformation of the ligand perfectly overlapped with the co-crystallized ligand, with an RMSD value of 0.33 Å and satisfactory precision in repositioning the LUR ligand within the active site of COX-2. The RMSD value between the docking pose and the crystallographic ligand pose is less than 2.0 Å (Figure 2).

The validated docking protocol was subsequently used for molecular docking simulation. Comparing the bindings of compounds 3 and 6 to the enzyme Hypoxia-inducible factor 1 (HIF1A), it is observed that compound 3 bound with lower energy and had a lower inhibition constant than 6. Regarding compounds 15 and 16 with Cyclooxygenase-2 (COX2), despite the low binding energy, the inhibition constants were higher than those of 3 and 6, with 16 being very high (Table 6).

**TABLE 6:** Values of the binding energies between the limonoids and HIF1A.

Molecules	Δ*E*_ele_	Δ*E*_vdW_	Δ*G*_GB_	Δ*G*_SA_	Δ*G*_bind_
YC-1	−10.18	−36.79	21.13	−4.62	−30.47
3	−19.07	−31.24	33.86	−4.09	−20.56
6	−11.69	−20.45	23.51	−2.69	−11.32

Caption: YC-1, lificiguat, 3- Methyl angolensate, 6 - 6-hydroxy-methyl angolensate.

**FIGURE 3:**
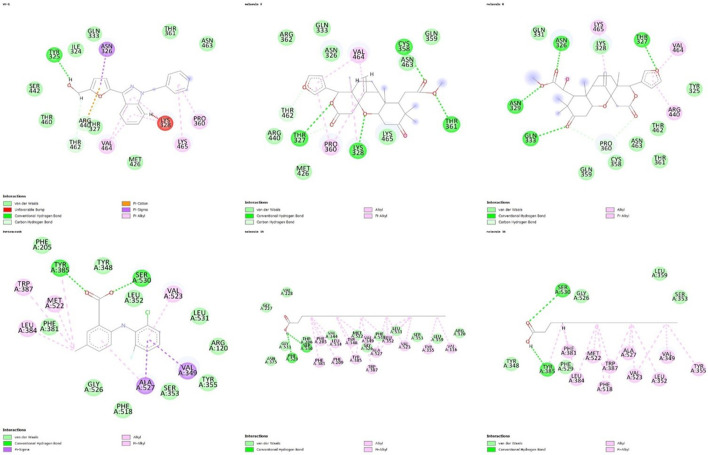
Representation of 2D interactions of molecules 3, 6, 15, 16 and inibidores YC-1, LUR. Image generated with Discovery Studio 3.5 Visualizer.

Regarding the interactions established between the limonoids and the HIF1A protein, compound 3 did not have any unfavorable bonds, establishing alkyl bonds and hydrogen bonding. Compound 6 presented 1 unfavorable bond, 1 alkyl bond, 1 C-H bond, and 4 hydrogen bonds (Figure 2). Evaluating the interactions established by the fatty acids and the COX-2 protein, unfavorable bonds are observed for both compounds, with hydrogen bonds, alkyl bonds, and C-H bonds also being observed (Figure 3).

### 3.3 Molecular dynamics simulation

#### 3.3.1 Interactions of the limonoids methyl angolensate and 6-hydroxy-methyl angolensate with HIF1A

**FIGURE 4:**
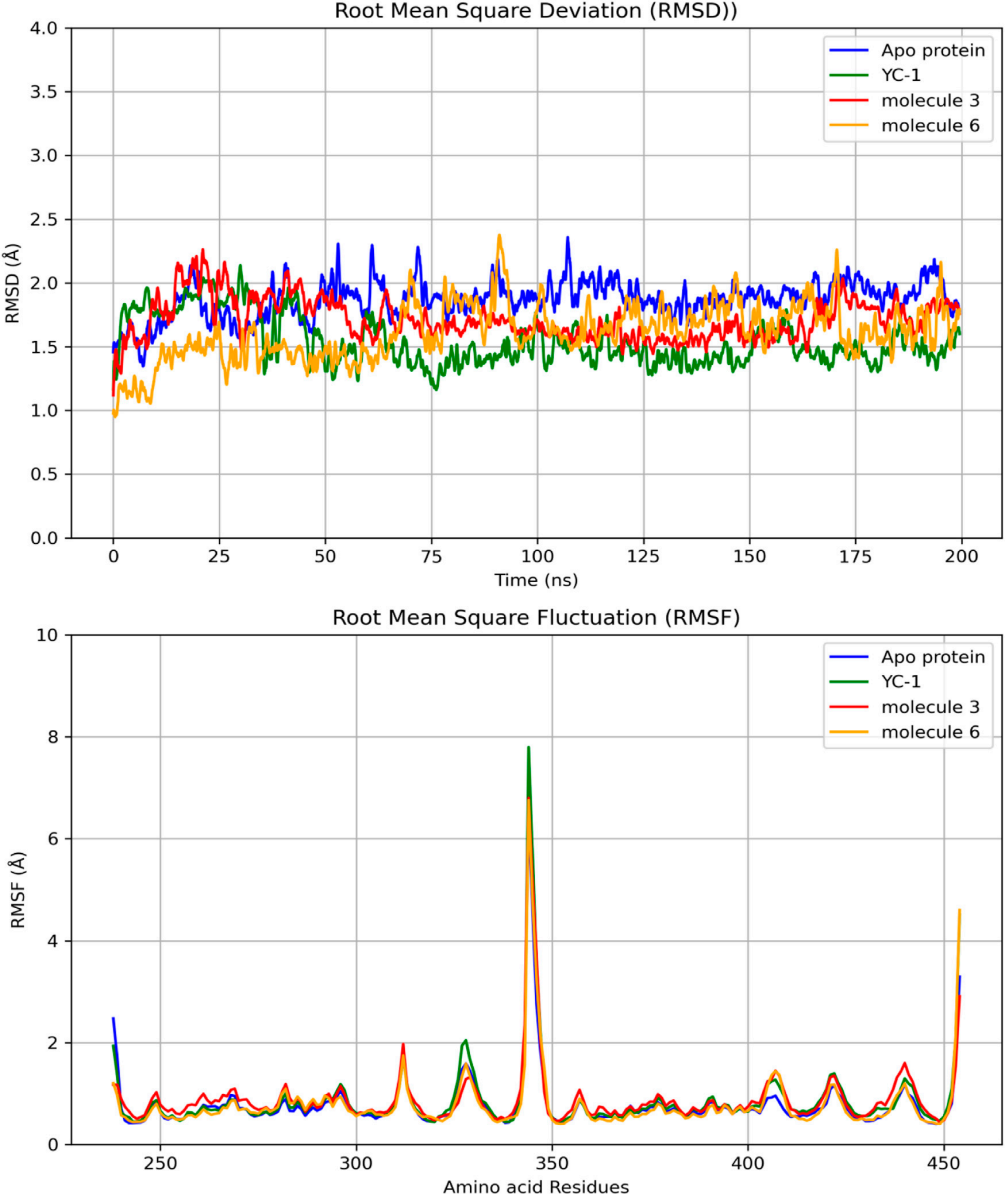
Comparisons of RMSD and RMSF of the limonoids and HIF1A as a function of time and amino acid residues. YC-1 - lificiguat, 3 - Methyl angolensate, 6 - 6-hydroxy-methyl angolensate.

Figure 4 shows the RMSDs of HIF1A complexed with ligands 3, 6, and YC-1, displaying stable dynamic behavior and RMSD values of 1.87 Å (molecule 3), 1.55 Å (molecule 6), 1.71 Å (YC-1), and 1.61 Å (HIF1A-6).

**FIGURE 5:**
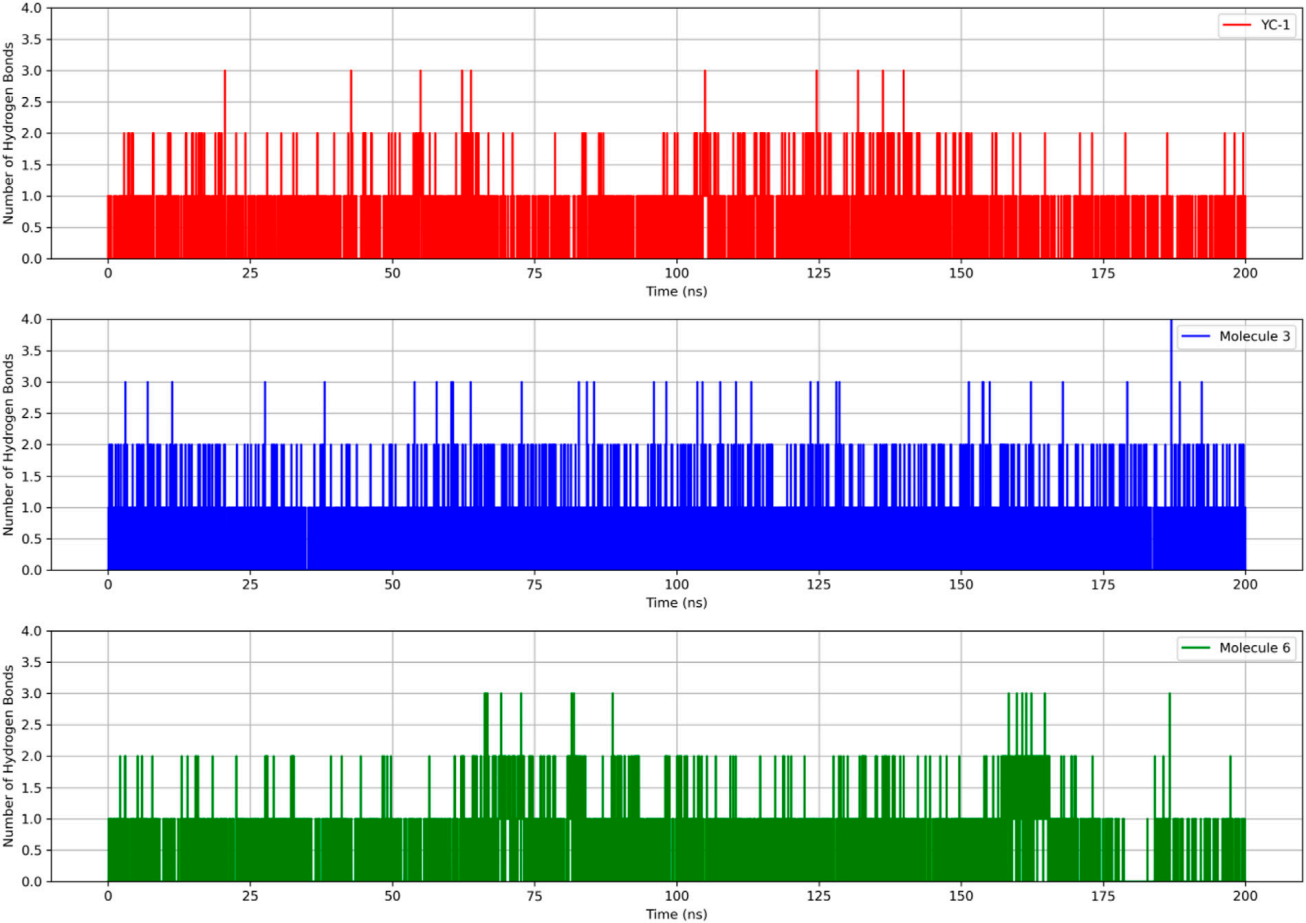
RMSD and hydrogen bonds between HIF1A and the YC-1 inhibitor and molecules 3 and 6.

Figure 5 shows that all complexes formed by molecules 3, 6, and the YC-1 inhibitor exhibited similar behaviors, with minimal fluctuations below 2 Å, except in the regions between residues 344-346, which showed greater fluctuation and the presence of a significant number of H bonds, suggesting a strong interaction between a ligand-protein complex.

In Table 6, it can be observed that molecule 3 showed the most favorable binding affinity to the HIF1A protein (ΔGbind −20.56 kcal/mol), compared to molecule 6 (ΔGbind −11.32 kcal/mol).

#### 3.3.2 Interactions of fatty acids with COX-2

**FIGURE 6:**
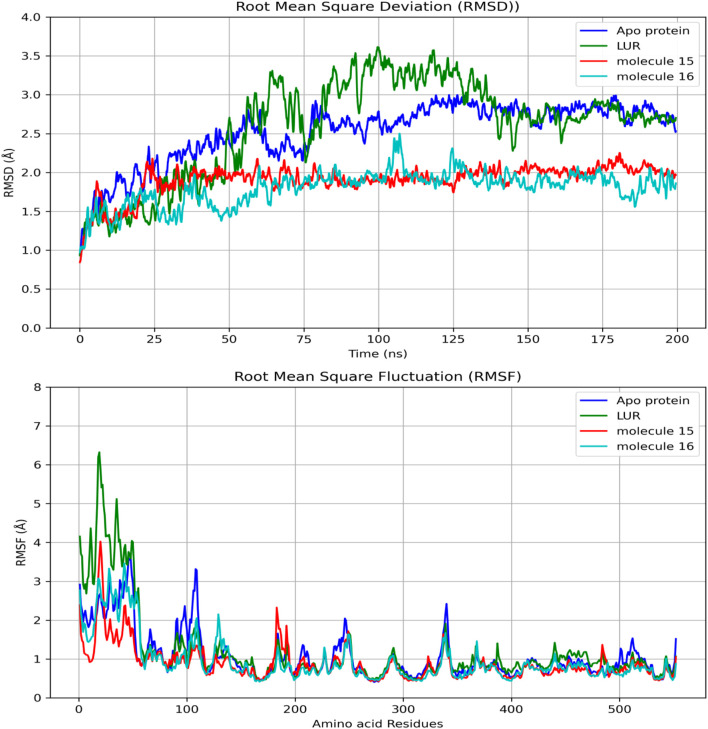
Comparisons of RMSD in the binding of fatty acids and COX-2 as a function of time and amino acid residues.

When comparing the RMSD values over time, it is observed that molecules 15 and 16 exhibit lower values than LUR (Figure 6). Regarding the comparison of RMSD and amino acid residues, in most bonds, proximities were observed between this parameter; however, the lowest RMSD values were observed for molecule 16 (Figure 6).

**FIGURE 7:**
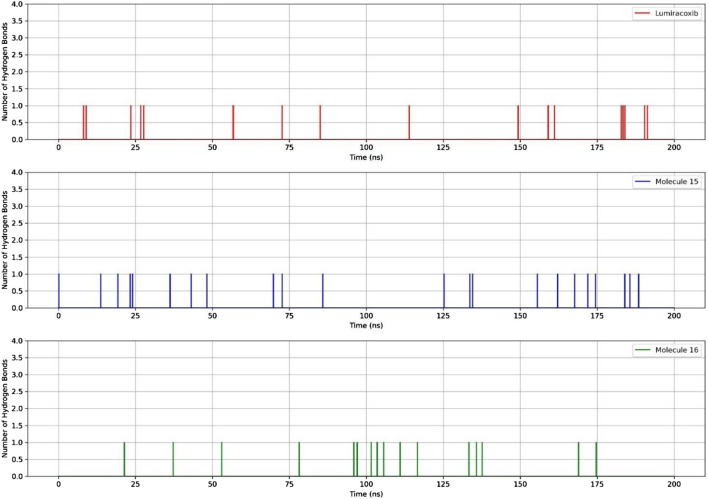
RMSD and hydrogen bonds between COX-2, LUR, and molecules 15 and 16.

Regarding the ligand’s ability to establish hydrogen bonds with COX-2, a greater number of bonds between the protein and molecule 15 were observed (Figure 7).

In Table 7, it can be observed that molecule 15 showed the most favorable binding affinity to the COX-2 protein (ΔGbind - 54.36 kcal/mol), compared to molecule 16 (ΔGbind - 35.90 kcal/mol).

**TABLE 7:** Values of the binding energies between fatty acids and COX-2.

Molecules	ΔEele	ΔEvdW	ΔGGB	ΔGSA	ΔGbind
LUR	−37.76	−35.77	44.42	−5.34	−34.46
15	−7.34	−59.96	21.55	−8.61	−54.36
16	−11.89	−40.82	22.82	−6.01	−35.90

Caption: LUR-lumiracoxib, 15 - Arachidic Acid, 16 - Myristic Acid.

## 4 Discussion

This study evaluated the physicochemical, pharmacokinetic, and toxicity aspects of fatty acids already identified in *C. guianensis* Oil, observing in the physicochemical study that they violate the LogP. The LogP assesses the balance between liposolubility and hydrosolubility, and when it is above 5, it can be a predictive factor for low absorption of the compounds in the gastrointestinal tract. However, pharmacokinetic prediction studies demonstrated that in MDCK cells, the permeability of the compounds was moderate to high, while in Caco2 cells, the permeability was moderate. The high permeability in MDCK cells suggests that these compounds may be absorbed by passive diffusion (Chen et al., 2018). That is, they can cross the lipid layer due to their high liposoluble potential. The permeability in Caco2 cells evaluates absorption in the Colon region, which seems to be moderate, and perhaps, the high intestinal absorption of these compounds may occur due to absorption in different locations of the GI tract (Da Silva Miranda et al., 2022).

Due to their MM < 500D and high liposolubility, the evaluated fatty acids appear to freely cross the blood-brain barrier. Therefore, therapeutic concentrations can be achieved centrally and peripherally, expanding their medicinal potential. However, adverse reactions may occur centrally and peripherally. Additionally, these compounds strongly bind to plasma protein and appear not to be metabolized by CYP. It is worth noting that phase 1 metabolism makes the compound more polar and facilitates renal excretion. It is important to emphasize that fatty acids play an essential role in the body, from strengthening immunity to their importance in the inflammatory response (Pereira, 2008).

A concerning point in terms of pharmacokinetics is the inhibitory potential of CYP2C19, CYP2C9, and CYP3A4, which may interfere with the metabolism of other drugs. Since CYP3A4 metabolizes a large number of drugs, its inhibition can lead to an increase in the plasma concentration of these drugs and elevate the risk of toxic effects.

Another important aspect evaluated was the toxicity of fatty acids in algae, crustaceans, and fish. All fatty acids were toxic to algae and crustaceans, while they were not toxic to fish. The model for algae is used to predict acute oral toxicity in terms of mortality (Guilhermino et al., 2000). The Daphnia crustacean model is used to predict acute and subchronic toxicities. The model for Medaka and Minnow fish suggests acute and subchronic toxicity, as well as changes in different organs (Bauer, 2017).

All fatty acids showed mutagenic potential (TA1535-NA), with mutations potentially occurring in both somatic and germline cells, depending on the genes, which may or may not have phenotypic effects, potentially leading to severe clinical consequences. Additionally, compounds 12, 17, 18, 19, and 20 were found to have carcinogenic potential in rats and mice, with carcinogenesis involving the conversion of a normal cell into a malignant cell, requiring prolonged time and repeated exposure to carcinogens (Loureiro et al., 2002). Thus, if used acutely or for short periods, the carcinogenic potential of fatty acids is minimized.

Regarding acute oral toxicity, the molecules with the lowest toxic potential are the fatty acids (Class V and VI). However, molecules 13, 14, and 16 appear to have side effects related to irritation, tumorigenicity, and mutagenicity. Therefore, while fatty acids may not be lethal when ingested, the side effects on organisms are a trade-off of these results, requiring attention to these molecules despite limited toxicity studies.

The limonoids, except for 11, followed the Lipinski rule; however, their permeability in MDCK cells showed that only one molecule had high permeability, suggesting that the mechanism used in cellular diffusion may not be passive diffusion (Chen et al., 2018). Additionally, the results in Caco2 cells showed moderate permeability, suggesting that absorption in the intestine occurs at more than one location, thus explaining the high intestinal absorption. However, limonoids have higher molecular mass (MM) compared to fatty acids, but only molecule 2 has a molecular mass (MM) exceeding 500D. On the other hand, molecule 11 violated the Lipinski rule. Despite limited oral bioavailability in molecules that do not adhere to Lipinski’s rule, the therapeutic potential should not be ignored (Lipinski, 2004).

Similarly to fatty acids, limonoids exhibited high intestinal absorption, despite low to moderate permeability in MDCK and moderate permeability in Caco2. These results suggest that perhaps the diffusion mechanism through membranes is not passive and that their absorption may occur in other intestinal regions (Chen et al., 2018). Another similarity with fatty acids was the potential inhibitory effect on CYPs, which could interfere with the metabolism of different classes of drugs (Chen et al., 2018).

In terms of toxicity, the significant advantage of limonoids over fatty acids is that they did not show mutagenic potential in predictions. A previous study demonstrated that limonoids found in andiroba oil have anti-inflammatory, anticancer, antitumor, and antiallergic properties (Matsui et al., 2014; Higuchi et al., 2017; Tsukamoto, 2019).

One disadvantage of limonoids compared to fatty acids was their higher acute oral toxicity, with their simulated LD50 belonging to class IV. However, it is important to establish the effective dose 50% of limonoids, thus allowing the determination of the therapeutic window of these compounds, ensuring their safety of use. On the other hand, there were no results related to side effects, which is encouraging for the possibility of a promising drug (Miranda-Júnior et al., 2012).

The molecular docking studies of the selected limonoids and fatty acids were conducted against molecular targets of Leishmania, aiming to explore their leishmanicidal potential. These enzymes are necessary for the parasite’s survival and represent relevant targets for the development of new drugs (Degrossoli et al., 2007). The limonoids exhibited the best characteristics and molecular affinities, as they formed hydrogen bonds with the Tyr254 residue, which participates in the active site, potentially generating irreversible inhibitors (Cardoso et al., 2012). Comparing the two limonoids and their binding to HIF1A, it can be suggested that limonoid 3 established a better binding.

Regarding fatty acids and their binding to cyclooxygenase 2, inhibition of which is related to anti-inflammatory effects, molecules 15 and 16 bound with favorable binding energy, but 16 had a very unfavorable inhibition constant. Thus, the more promising molecule was 15, which may contribute to the treatment of cutaneous leishmaniasis in the wound healing phase. This process involves interaction between cells and various messenger systems, divided into three phases: inflammatory, proliferative, and remodeling (Velnar et al., 2009).

The results of molecular dynamics provide a detailed and dynamic view of molecular behavior, essential for understanding complex phenomena of molecule-protein binding. Despite the RMSD values of limonoids 3 and 6 being close and many hydrogen bonds being observed for both molecules, the better binding energy was observed for limonoid 3, suggesting that it may be the most promising.

In terms of the dynamics of fatty acids 15 and 16, it was observed that the RMSD of these molecules was lower than that of LUR. However, there was a slight difference between the number of hydrogen bonds and the energy, with compound 15 being the most promising.

## 5 Conclusion

In summary, the leishmanicidal effect of *C. guianensis* appears to result from the synergistic effect between limonoids and fatty acids. Limonoids have an antiparasitic effect, while fatty acids may contribute to the wound healing process of American cutaneous leishmaniasis. Another relevant point is related to mutagenicity, with only fatty acids presenting this potential, while limonoids act as protectors against mutagenic processes. Therefore, *C. guianensis* oil seems to be very promising for the treatment of cutaneous leishmaniasis.

## Data Availability

The original contributions presented in the study are included in the article/Supplementary Material, further inquiries can be directed to the corresponding author.
